# First detection and molecular characterization of influenza D virus in livestock from Kazakhstan reveals circulation of the D/Yama2019 lineage

**DOI:** 10.3389/fvets.2026.1911871

**Published:** 2026-08-26

**Authors:** Yermukhammet Kassymbekov, Temirlan Sabyrzhan, Sardor Nuralibekov, Yersultan Shayakhmet, Sumbat Suleimenova, Yelizaveta Khan, Marat Kumar, Kobey Karamendin, Aidyn Kydyrmanov

**Affiliations:** Research and Production Center for Microbiology and Virology, Almaty, Kazakhstan

**Keywords:** cattle, Central Asia, HEF gene, influenza D virus, Kazakhstan, phylogenetics, surveillance

## Abstract

Influenza D virus (IDV) is an emerging *Orthomyxovirus* primarily associated with cattle and increasingly recognized as a pathogen of veterinary and zoonotic relevance. Despite growing evidence of its circulation across Asia, Europe, and North America, no data have previously been available from Kazakhstan or Central Asia. This study investigated the presence of IDV in domestic livestock samples collected during active surveillance between 2023 and 2026. A total of 867 biological samples were obtained from cattle (*n* = 611), camels (*n* = 175), Maral Deer (*n* = 51), and pigs (*n* = 30) across multiple regions of Kazakhstan. Viral RNA was screened by reverse transcription polymerase chain reaction (RT-PCR) targeting the *HEF*, *PB1*, *PB2*, and *P3* genes using a newly designed primer panel. Positive amplicons were subjected to Sanger sequencing and phylogenetic analysis. IDV RNA was detected in 11 animals, corresponding to an overall prevalence of 1.27%. Positive samples were identified in several geographically distinct regions, indicating wider circulation than previously recognized. Complete *HEF* gene sequences were successfully obtained from two representative cattle strains, designated D/bovine/Zhetysu/KAZ/678/2025 and D/bovine/Zhetysu/KAZ/680/2025. Phylogenetic analysis demonstrated that both strains clustered within the D/Yama2019 lineage and shared >98.83% nucleotide identity with contemporary Asian strains. To our knowledge, this is the first report of IDV detection in Kazakhstan. These findings expand the known geographic range of IDV and suggest possible transboundary transmission routes across Central Asia. Continued molecular surveillance is warranted to clarify epidemiology, host range, and potential economic impact.

## 1 Introduction

Influenza D virus (IDV) is a recently recognized member of the family *Orthomyxoviridae*, officially classified within the distinct genus *Deltainfluenzavirus* in 2016 (1–3).

Influenza viruses are among the most important viral pathogens affecting humans and animals because of their high evolutionary potential, ability to cross species barriers, and capacity to establish infections in diverse host species. Unlike several domestic animal species that harbor host-adapted influenza A viruses, cattle are not known to possess a species-specific influenza A virus lineage. Instead, growing evidence indicates that bovines serve as the primary reservoir of IDV and play a central role in its maintenance, evolution, and transmission to other domestic and wild mammals (4, 5).

First identified in 2011 in swine in Oklahoma, USA, IDV is now known to predominantly circulate in cattle (6–8). In cattle, IDV infection is commonly associated with mild to moderate respiratory disease and contributes to the bovine respiratory disease complex (BRDC), resulting in considerable economic losses to the global dairy and beef industries (3, 6, 9).

IDV exhibits broad host tropism, including swine, sheep, goats, camels, horses, dogs, cats, and wild ungulates (3, 4, 10, 11). Emerging evidence of IDV-specific antibodies in humans, particularly veterinarians and livestock workers, and the ability of the virus to replicate in primary human airway epithelial cell cultures, suggest potential zoonotic risk that remains to be fully established (5, 12, 13). Under the One Health concept, global IDV surveillance is increasingly important for assessing interspecies transmission risks and potential adaptation to human populations (14).

IDV genetic diversity is primarily classified based on the hemagglutinin–esterase–fusion (*HEF*) gene, which mediates receptor binding and is the primary target of neutralizing antibodies (6, 15). At least five major lineages are recognized: D/OK, D/660, D/Yama2016, D/Yama2019, D/CA2019, and D/France (2, 3, 6). While D/OK and D/660 has wide geographic distribution, the D/Yama2019 lineage, originally identified in Japan, has emerged as the dominant lineage in Asia in recent years, with reports in China, Türkiye, South Korea and more recently in Australia (7, 16). High nucleotide substitution rates and frequent reassortment drive rapid evolution and expanding geographic distribution of IDV (7, 8, 17).

Respiratory diseases remain a major cause of economic losses in cattle production worldwide and are typically associated with the multifactorial BRDC, which involves interactions among viral and bacterial pathogens, environmental stressors, and host factors. Although several important cattle pathogens, including bovine herpesvirus 1 (BoHV-1), bovine coronavirus (BCoV), and bovine viral diarrhoea virus (BVDV), have recently been reported in Kazakhstan, comprehensive surveillance data on respiratory viruses in the country remain limited. Recent nationwide studies have confirmed the circulation of these pathogens in bovine herds across Kazakhstan, highlighting the need for expanded molecular surveillance of emerging respiratory viruses and their contribution to cattle health (18, 19).

Despite widespread IDV circulation across Asia and the emergence of D/Yama2019 in neighboring countries, there are no published reports of molecular identification of IDV in Kazakhstan livestock. This study provides the first nationwide molecular detection and HEF-based lineage characterization of IDV in the Kazakhstan livestock, addressing a critical gap in regional IDV surveillance (20). Here, we report the molecular detection and lineage characterization of IDV in Kazakhstan cattle, providing the first national-scale evidence of IDV circulation and genetic diversity in the country.

## 2 Materials and methods

### 2.1 Sample collection and ethics statement

Between January 2023 and March 2026, nasopharyngeal samples were collected from cattle, camels, pigs and farmed Altai Maral Deer (*Cervus elaphus sibiricus*) during routine veterinary surveillance programs in six regions of Kazakhstan: Zhetysu, East Kazakhstan, Mangystau, Aktobe, Zhambyl and Almaty.

Biological specimens were obtained from apparently healthy animals, as well as from individuals exhibiting clinical respiratory signs such as nasal discharge and coughing, as documented in the accompanying field records. Full details of all specimens collected, including sampling locations, farm identifiers, host attributes, total sample counts and positivity rates, are provided in Supplementary Table S1.

Nasopharyngeal swabs were collected using sterile cotton-tipped applicators and immediately immersed in a viral transport medium containing penicillin (2,000 U/mL), streptomycin (2 mg/mL), gentamicin (50 μg/mL), nystatin (50 U/mL) and 0.5% bovine serum albumin (21). Samples were maintained in liquid nitrogen (−196 °C) during field collection and transportation and were subsequently stored at −80 °C until laboratory analysis.

No experimental infections, invasive procedures, or direct animal manipulations were performed specifically for this study. The study protocol was reviewed and approved by the Ethics Committee of the Research and Production Center for Microbiology and Virology (protocol no. 02-09/190 dated 30 October 2023).

### 2.2 RNA extraction and real-time RT-PCR screening for influenza D virus

Total viral RNA was extracted from 140 μL of sample suspension using the QIAamp Viral RNA Mini Kit (Qiagen GmbH, Hilden, Germany) according to the manufacturer’s instructions. RNA was eluted in 60 μL of RNase-free water and stored at −80 °C until molecular analysis.

All RNA extracts were screened for IDV using a RT-qPCR assay targeting the polymerase basic 1 (PB1) gene. Amplification was performed using the Luna® Universal One-Step RT-qPCR Kit (New England Biolabs, USA) according to the manufacturer’s recommendations. The assay employed the following oligonucleotides: forward primer 5′-TGGATGGAGAGTGCTGCTTC-3′, reverse primer 5′-GCCAATGCTTCCTCCCTGTA-3′, and TaqMan probe 5′-FAM-CATGTTAAACATTCCCATCAGCATTCCT-BHQ1-3′. Primers and probe were designed based on conserved regions of the IDV PB1 gene (22).

Real-time RT-qPCR was performed on a QuantStudio 5 Real-Time PCR System (Applied Biosystems, Foster City, CA, USA). Thermal cycling conditions consisted of reverse transcription at 50 °C for 20 min, initial denaturation at 95 °C for 5 min, followed by 45 cycles of 94 °C for 15 s and 60 °C for 60 s. Fluorescence data were acquired during each annealing/extension step.

Each run included a positive control containing IDV RNA and a no-template negative control.

### 2.3 Primer design for influenza D virus genome sequencing

For subsequent molecular detection and to ensure the generation of amplicons suitable for downstream sequencing and phylogenetic analysis, a primer panel targeting the *HEF*, *PB1*, *PB2*, and *P3* gene segments of IDV was designed.

The primer panel presented in Table 1 was developed based on the alignment of more than 100 complete IDV genome sequences retrieved from international databases, ensuring broad applicability across all known genetic lineages. Sequence alignment and comparative analysis were performed using the Geneious software platform, which enabled the identification of regions with minimal variability and stable primer binding sites. Oligonucleotide design was carried out using the built-in tools of Geneious Prime, with additional optimization performed in the Primer3Plus online platform, taking into account primer length, GC content, melting temperature, absence of significant secondary structures, and potential primer–dimer formation.

**Table 1:** List of synthesized primer panel, including target regions and primer sequences for the *HEF*, *PB1*, *PB2*, and *P3* genes of Influenza D virus.

Segment	Primer name	Sequence (5′ → 3′)	Target region (nt)
HEF	HEF-1-F1	AGCATAAGCAGGAGATTTTCAAAGAR	1–1,100
	HEF-1-R1	CCTCCATCAGTGTCGAAGCA	
	HEF-1-F2	TGCAAAGAGTGAATGAAAGCTTCT	
	HEF-1-R2	ACATCCTTCTGTTTGTAGGCA	
	HEF-2-F1	TGTCCTTCCAAAATCTTGAACCC	900–2,053
	HEF-2-R1	CAACCTTGGTGTCAACTGGC	
	HEF-2-F2	CTGAGTGGTCAGCTTCACGA	
	HEF-2-R2	AGCAGCAGTAGCAAGGAGAT	
PB1	PB1-1-F1	GGCATAAGCAGAGGATTTTATAACA	1–1,300
	PB1-1-R1	TTCCCATCAGCATTCCTCCC	
	PB1-1-F2	ACCCATATCTACTCATGTTAAACAATG	
	PB1-1-R2	TCATGACTCCAAAAACAGTTGACA	
	PB1-2-F1	TAGTGATACCCGCAAAAAGGA	1,100–2,331
	PB1-2-R1	GCTTGGCGTCCTCGATTAGT	
	PB1-2-F2	TGGAGGGACCTGTTTGAAACA	
	PB1-2-R2	GCAGTAGCAAGAGGATTTTTCTGTTAT	
PB2	PB2-1-F1	GGCATAAGCAGAGGATGTCACT	1–1,400
	PB2-1-R1	GTCTCTCGATTGCCTTACCCA	
	PB2-1-F2	CGCTCGCAAAAGAGTATGCA	
	PB2-1-R2	AGGCATCCAAGTTGTCTCTCG	
	PB2-2-F1	ACAAGAGAAATGAGTTGGAGCCT	1,200–2,364
	PB2-2-R1	TCAAAGTACTTAGCTCCATTGGACT	
	PB2-2-F2	ACAGGAGGACAAATGGCAAGA	
	PB2-2-R2	AGCAGTAGCAAGGAGATTTTTAACA	
P3	P3-1-F1	GGCATAAGCAGGAGATTTAGAAATGT	1–1,200
	P3-1-R1	TCCTCTCTGGATTCTTGCCA	
	P3-1-F2	ACATCGCTGAAGAAGTGGTCA	
	P3-1-R2	CTCATTGCCCAAAGCCATTCT	
	P3-2-F1	AGGACAAGAGAAATGAGTTGGAG	1,000–2,228
	P3-2-R1	GATTTTTAACATTACAAGGCCTTTGGT	
	P3-2-F2	TGAGTTGGAGCCTTCAGAACA	
	P3-2-R2	AGCAGTAGCAAGGAGATTTTTAACA	

The designed primers were synthesized by Macrogen (Seoul, South Korea; Table 1) using standard oligonucleotide chemical synthesis methods described in the literature (23, 24).

The subsequent step involved complementary DNA (cDNA) synthesis followed by amplification of the target gene fragment using RT-PCR. Reverse transcription was performed using the ProtoScript II First Strand cDNA Synthesis Kit (New England Biolabs, USA) according to the manufacturer’s instructions. The resulting cDNA was used as a template for downstream PCR amplification.

Samples with Ct values below 34 were considered positive. Samples with borderline amplification were re-tested in duplicate and classified according to laboratory validation criteria.

Amplification was carried out using the high-fidelity enzyme mix Q5 Hot Start High-Fidelity 2 × Master Mix (New England Biolabs, USA), which ensures high reaction specificity and a low replication error rate. The reaction mixture contained 1 × master mix, forward and reverse HEF primers at working concentrations, template cDNA, and nuclease-free water to the final reaction volume. The PCR thermal cycling conditions included an initial denaturation at 95 °C for 3 min, followed by 35 cycles consisting of 20 s at 94 °C, 30 s at 60 °C for primer annealing, and 60 s at 72 °C for extension, with a final extension step at 72 °C for 5 min.

The resulting amplicons were visualized by electrophoresis on a 2% agarose gel and used for further analysis.

### 2.4 Virus isolation on cell culture

For IDV isolation, nasopharyngeal swabs from cattle that tested positive by PCR were filtered through a 0.22 μm membrane and treated with antibiotics (penicillin, streptomycin, amphotericin B) prior to inoculation into Madin-Darby Canine Kidney (MDCK) and Madin-Darby Bovine Kidney (MDBK) cell lines. The inoculated cells were incubated at 37 °C in a 5% CO₂ atmosphere. Three serial passages were performed to facilitate virus isolation. The cells were monitored daily for signs of viral infection by visually inspecting them for cytopathic effects (CPE), performing RT-PCR assays, and conducting hemagglutination tests using 0.75% turkey red blood cells.

### 2.5 Genome sequencing and phylogenetic analysis

Target PCR products were purified using a QIAGEN purification kit to remove residual primers, unincorporated deoxynucleotide triphosphates (dNTPs), enzymes, and other reaction components, according to the manufacturer’s protocol. The purified amplicons were used as templates for cycle sequencing reactions. PCR products were selected for sequencing if they produced a single dominant band of the expected size and yielded sufficient DNA for subsequent analysis. Amplicons showing low yield or non-specific amplification were not sequenced. The selected products were then purified and sequenced bidirectionally using the Sanger method.

Sequencing reactions were performed using the BigDye Terminator sequencing chemistry kit, which contains fluorescently labelled chain-terminating nucleotides, in accordance with the manufacturer’s recommendations. Upon completion of the sequencing reaction, the products were further purified using the BigDye XTerminator Purification Kit to remove unincorporated dyes and reaction by-products.

Raw Sanger sequencing chromatograms were inspected, trimmed, and assembled into consensus sequences using BioEdit v7.2.5. Sequence quality was assessed manually, and ambiguous nucleotide positions were verified by examination of forward and reverse chromatograms.

The resulting consensus sequences were compared with publicly available IDV sequences using the BLASTn tool available through the National Center for Biotechnology Information (NCBI) database to determine their genetic relatedness and lineage affiliation.

Reference *HEF* gene sequences representing the major recognized IDV lineages, including D/swine/Oklahoma/1334/2011 (D/OK), D/bovine/Oklahoma/660/2013 (D/660), D/bovine/Yamagata/10710/2016 (D/Yama2016), D/bovine/Yamagata/1/2019 (D/Yama2019), and D/bovine/California/0363/2019 (D/CA2019), together with representative contemporary strains from China (D/bovine/CHN/JY3125/2022 and D/bovine/CHN/JY3001/2021), were retrieved from the GenBank database for comparative analyses.

Multiple sequence alignment was performed using the ClustalW algorithm implemented in MEGA version 11. Phylogenetic relationships were inferred using the maximum-likelihood method under the Tamura–Nei nucleotide substitution model with 1,000 bootstrap replicates. The resulting phylogenetic tree was used to determine the evolutionary placement of the Kazakhstan IDV strains relative to established global lineages.

The sequence data generated in this study have been deposited in the GenBank database under the following accession numbers: HEF gene segment: PZ440420-PZ440421, and P3 gene segment: PZ712063-PZ7120644.

## 3 Results

A total of 867 biological samples were collected and analyzed during the study period. The sampled population comprised cattle (*n* = 611), camels (*n* = 175), farmed Altai Maral Deer (*Cervus elaphus sibiricus*; *n* = 51), and pigs (*n* = 30). Samples were obtained from animals from six regions of Kazakhstan: Zhetysu, East Kazakhstan, Mangystau, Aktobe, Zhambyl, and Almaty.

IDV RNA was detected in two host species with varying prevalence rates. Among cattle, 9 out of 611 animals tested positive, corresponding to a detection rate of 1.47%. In the overall sample set, 11 of 867 animals were positive, yielding a prevalence of 1.27% (Table 2).

**Table 2:** Distribution of collected samples and detection of influenza D virus in livestock and farmed ungulates in Kazakhstan, 2023–2026.

Animal species	Number of samples (*n*)	Proportion (%)	PCR-positive (*n*)	Positivity rate (%)
Cattle (*Bos taurus*)	611	70.5	9	1.47
Camel (*Camelus bactrianus*)	175	20.2	0	0
Altai maral (*Cervus elaphus sibiricus*)	51	5.9	2	3.92
Pigs *Sus scrofa domesticus*	30	3.4	0	0
Total	867	100	11	1.27

Notably, a higher detection rate was observed in farmed Altai Maral Deer, with 2 of 51 animals testing positive (3.92%). Both positive animals were clinically healthy at the time of sampling. Maral Deer in Kazakhstan are commonly maintained in fenced, free-ranging pastures for antler production and may share grazing areas with cattle, creating opportunities for direct or indirect interspecies exposure. Attempts to obtain *HEF* gene sequences from the positive Maral Deer samples were unsuccessful due to low viral RNA concentrations (Ct values >34), thereby precluding further genetic characterization.

Three IDV-positive samples were subjected to virus isolation attempts in MDCK and MDBK cell cultures. Following inoculation and three serial passages, no cytopathic effects were observed in either cell line. RT-PCR screening of cell culture supernatants detected a weak positive signal for sample D/bovine/Zhetysu/KAZ/678/2025 after the first passage; however, viral RNA was not detected in subsequent passages, indicating the absence of sustained viral replication. Consequently, no viable IDV isolate was recovered from the positive samples.

These findings indicate the presence of IDV at a low prevalence within livestock populations in Kazakhstan. Nevertheless, due to uneven sampling sizes among different host species, comparisons of prevalence rates should be interpreted with caution.

Positive animals were identified in several geographically distinct regions of Kazakhstan, including: Zhetysu, Aktobe, Mangystau, and Zhambyl. These findings indicate that IDV circulation is not restricted to a single outbreak focus but may be geographically widespread.

No statistically meaningful conclusions could be drawn for the minor sampled species due to limited sample sizes.

As a result, a primer panel was generated to amplify overlapping fragments spanning the entire HEF gene segment, thereby increasing the reliability of molecular detection of IDV and ensuring the production of amplicons suitable for subsequent sequencing and phylogenetic analysis. Complete *HEF* gene sequences were successfully obtained from two cattle samples positive for IDV: D/bovine/Zhetysu/KAZ/678/2025 and D/bovine/Zhetysu/KAZ/680/2025. These sequences from Kazakhstan exhibited high nucleotide similarity and formed a close phylogenetic cluster with other Asian IDV strains.

**Figure 1:**
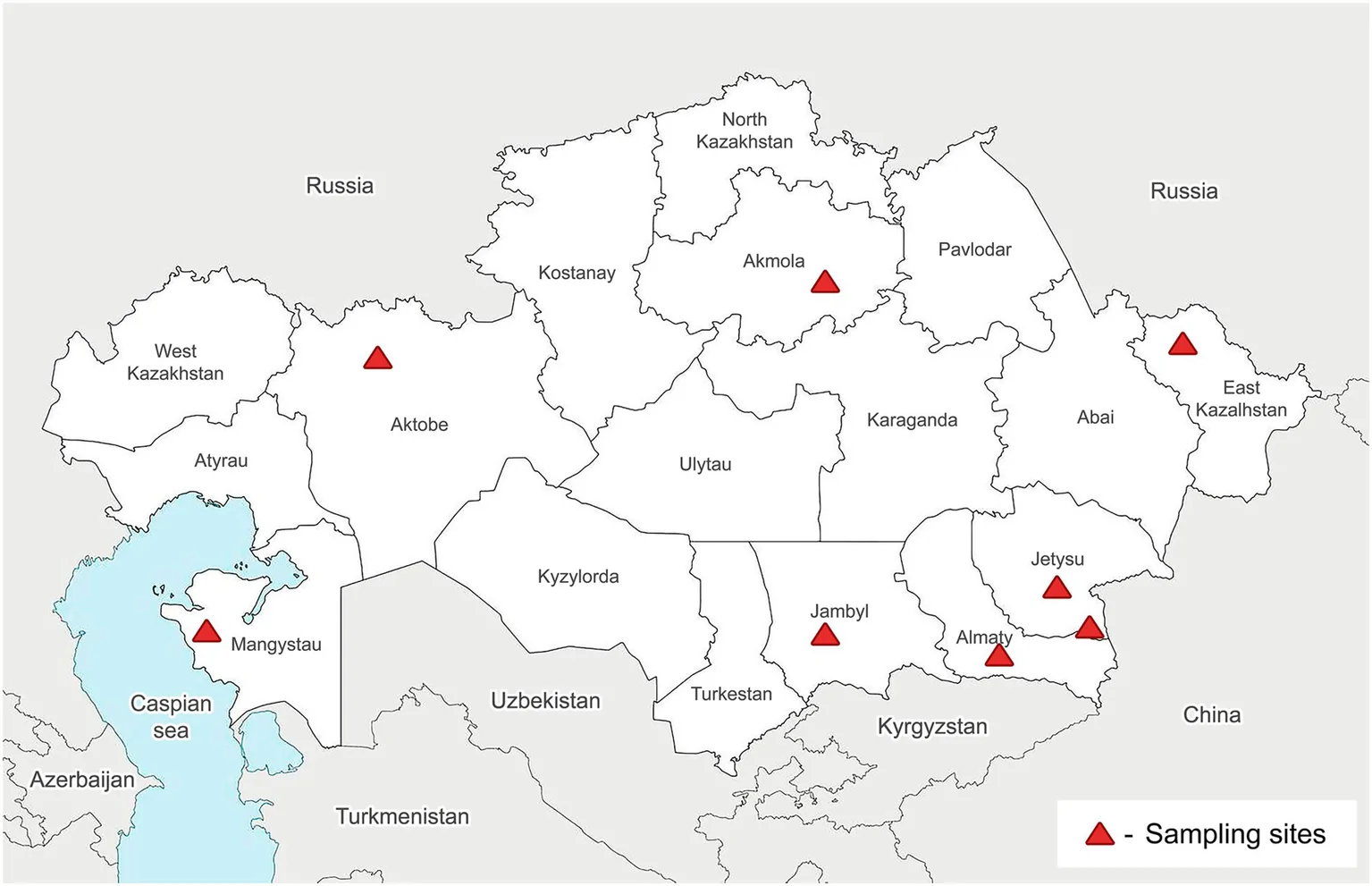
Geographic distribution of sampling sites. Red triangles indicate the regions where animal samples were collected.

To evaluate the genetic relatedness of these IDV strains to previously characterized HEF sequences, the obtained sequences were aligned with representative strains from various lineages. The alignment results indicated that the *HEF* sequences of D/bovine/Zhetysu/KAZ/678/2025 and D/bovine/Zhetysu/KAZ/680/2025 exhibited a high degree of similarity to the *HEF* sequence of the D/Yama2019 lineage, with nucleotide identities exceeding 98.83%. Consequently, the *HEF* sequences of these strains clustered phylogenetically within the D/Yama2019 lineage, as illustrated in Figure 1.

**Figure 2:**
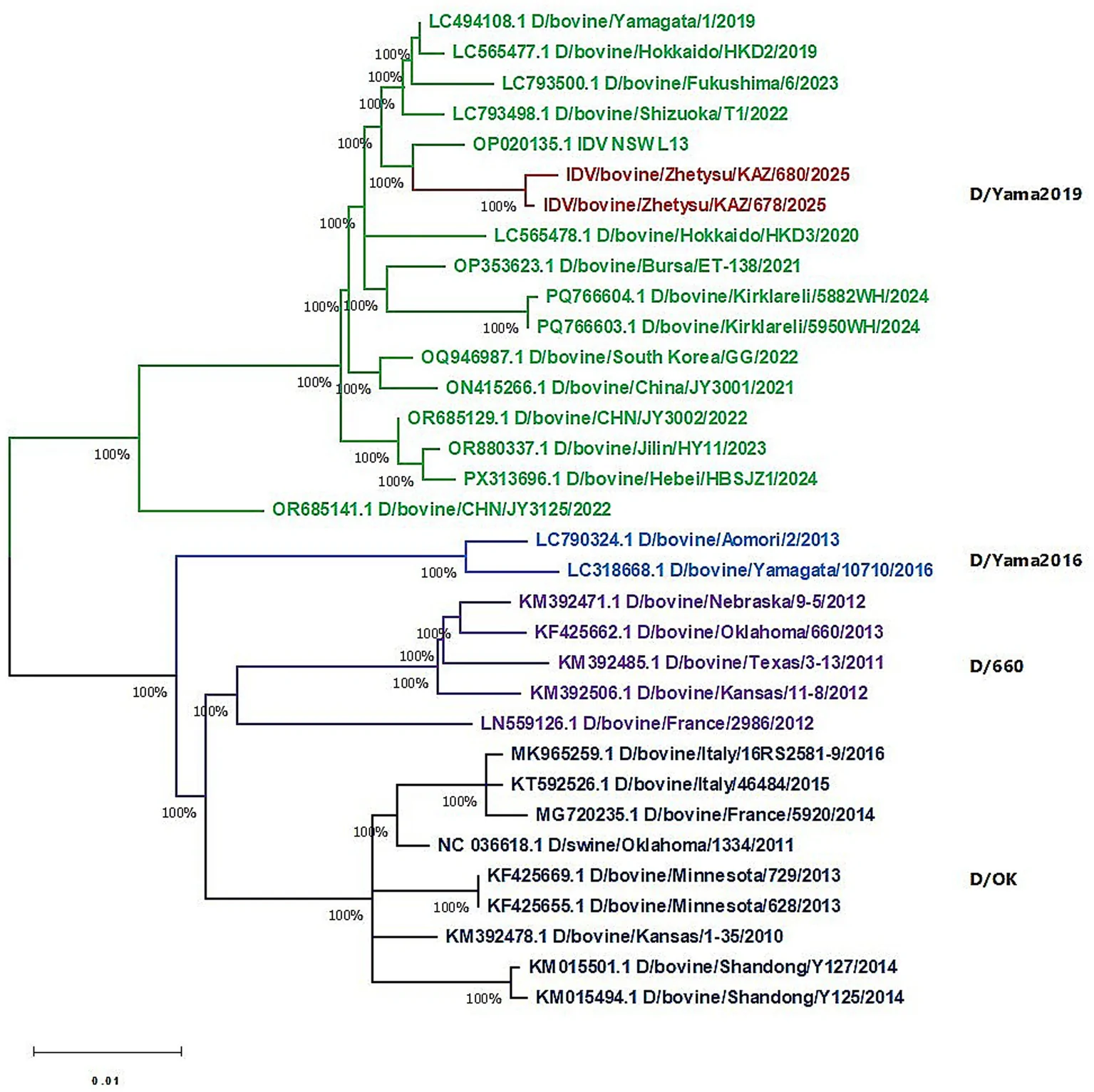
Phylogenetic tree of the HEF gene nucleotide sequences of Influenza D viruses D/bovine/Zhetysu/KAZ/678/2025 and D/bovine/Zhetysu/KAZ/680/2025, together with related viruses isolated from domestic animals between 2011 and 2024, retrieved from the GenBank database.

The evolutionary history was inferred by using the Maximum Likelihood method and Tamura-Nei model (25). The tree with the highest log likelihood (−5,582.92) is shown. The percentage of trees in which the associated taxa clustered together is shown below the branches. Initial tree(s) for the heuristic search were obtained by applying the Neighbor-Joining method to a matrix of pairwise distances estimated using the Tamura-Nei model. A discrete Gamma distribution was used to model evolutionary rate differences among sites [5 categories (+*G*, parameter = 0.3684)]. The tree is drawn to scale, with branch lengths measured in the number of substitutions per site. The proportion of sites where at least 1 unambiguous base is present in at least 1 sequence for each descendant clade is shown next to each internal node in the tree. This analysis involved 33 nucleotide sequences. Codon positions included were 1st + 2nd + 3rd + Noncoding. There were a total of 1866 positions in the final dataset. Evolutionary analyses were conducted in MEGA11 (26) (Figure 2).

**Figure 3:**
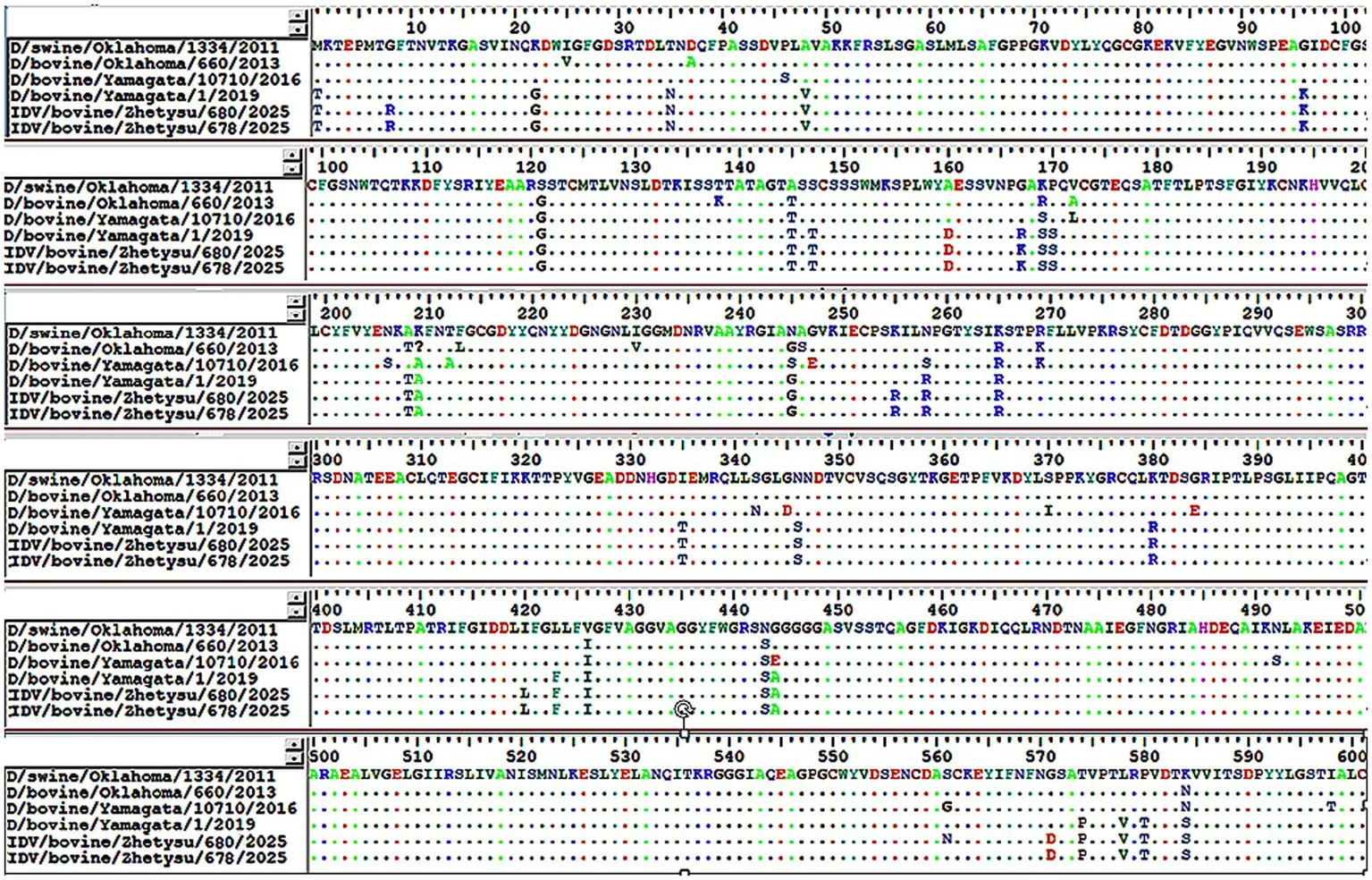
Multiple amino acid alignment of HEF proteins of Kazakhstan Influenza D virus strains and representative global reference lineages.

Comparative amino acid analysis was performed for the *HEF* proteins of two Kazakhstan IDV variants D/bovine/Zhetysu/KAZ/678/2025 and D/bovine/Zhetysu/KAZ/680/2025 (Figure 3).

The Kazakhstan *HEF* sequences encoded complete open reading frames corresponding to proteins of approximately 621 amino acids, consistent with previously described functional *HEF* glycoproteins of IDV. No premature stop codons, frameshift-associated truncations, or major deletions were identified, indicating that the *HEF* protein remains structurally intact.

Analysis of the receptor-binding domain (RBD) revealed conservation of critical amino acid residues involved in the recognition of 9-*O*-acetylated sialic acid receptors. Several residues associated with receptor engagement and stabilization of the receptor-binding cavity were highly conserved across both Kazakhstan strains, including residues corresponding to positions 185, 229, 231, 239, and 273.

The hydrophobic fusion-associated region of *HEF* was also fully conserved. The characteristic fusion motif, FIGFVAGGVAGGYF, was identical across the Kazakhstan strains and contemporary reference strains, suggesting preservation of membrane-fusion functionality and viral entry capacity.

Conserved esterase-associated residues involved in receptor-destroying activity were also maintained, supporting retention of functional *HEF* enzymatic properties.

Special emphasis was placed on the apex region of the *HEF* protein (residues 210–214), recognized as a key lineage-defining and antigenically significant domain of the IDV.

**Figure 4:**
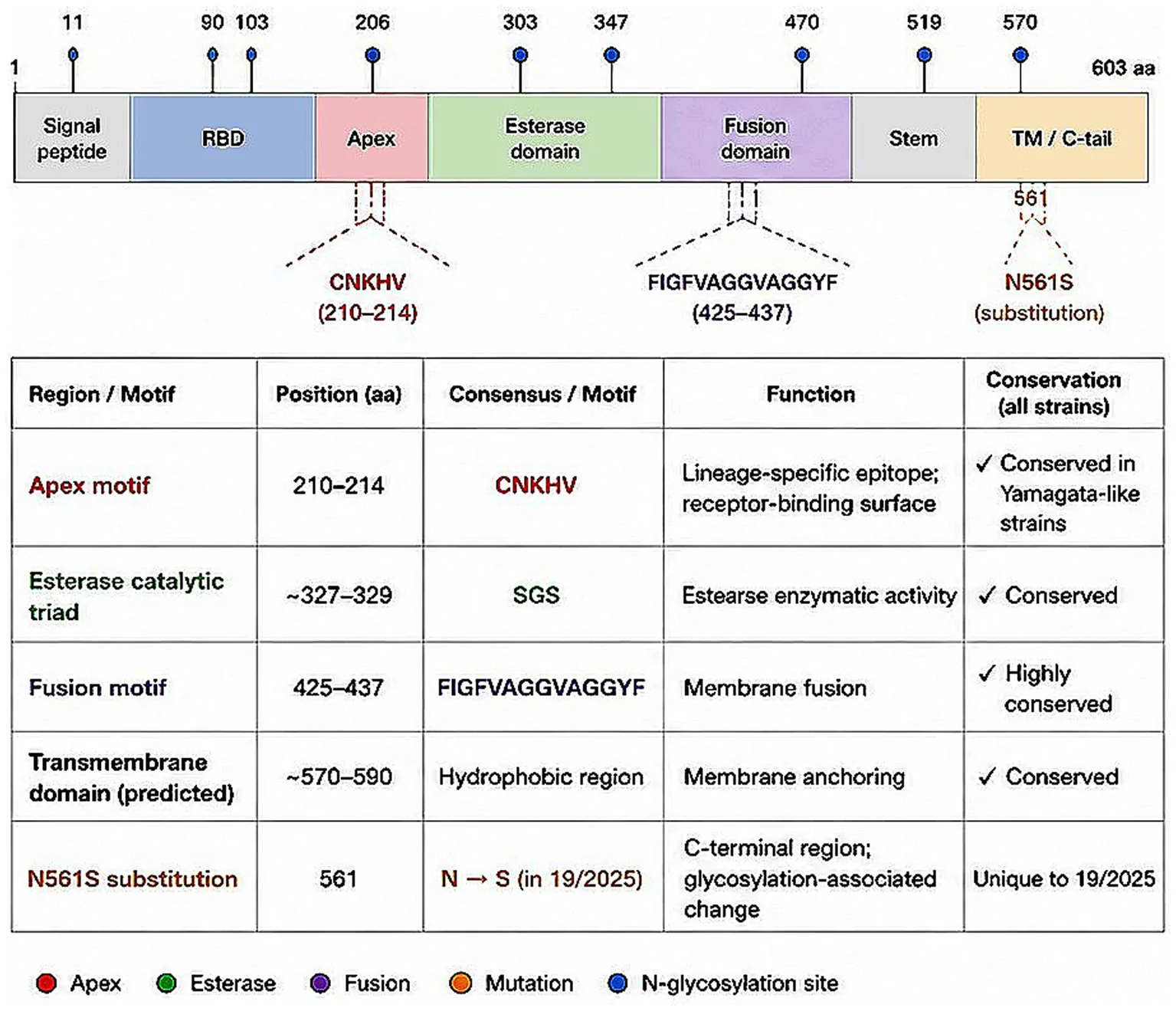
Conserved HEF functional regions and lineage-associated molecular signatures supporting D/Yama2019-like affiliation of Kazakhstan Influenza D virus strains.

Kazakhstan strains clustered with D/Yama2019 and possessed a conserved CNKHV apex motif (Figure 4).

A comparative analysis revealed that this motif exhibits the highest similarity to contemporary D/Yama2019-like viruses and diverges from the classical D/OK and D/660 lineages that have traditionally been predominant in North America.

The observed apex profile supports phylogenetic affiliation of the Kazakhstan strains with the emerging Yamagata-related lineage currently circulating in East Asia.

The amino acid sequences of IDV/bovine/Zhetysu/678/2025 and IDV/bovine/Zhetysu/680/2025 exhibit a remarkable degree of conservation throughout the entire *HEF* coding region.

Only a single amino acid substitution was identified in the C-terminal region *N* → S. This substitution may influence a potential *N*-glycosylation motif and could contribute to minor antigenic or structural variability. Nevertheless, the overall degree of divergence between the two Kazakhstan strains remained exceptionally low, suggesting recent local circulation of a genetically conserved viral population.

Multiple putative N-glycosylation motifs (N-X-S/T) have been identified throughout the *HEF* protein. These glycosylation-associated motifs are regarded as critical determinants influencing antigenic masking, immune evasion, receptor accessibility, and host adaptation.

One of the Kazakhstan strains preserved a potential glycosylation-associated motif in the C-terminal region, which was modified in the second strain as a result of the observed *N* → *S* substitution.

A comparative analysis of the *P3* gene was conducted on the IDV sequences D/bovine/Zhetysu/KAZ/678/2025 and D/bovine/Zhetysu/KAZ/680/2025 from Kazakhstan, using representative global IDV reference sequences, including those related to D/Yama2019. The sequences obtained for *P3* encoded complete open reading frames, with no evidence of frameshift mutations, premature stop codons, or significant deletions, thereby indicating the preservation of structurally intact polymerase proteins.

Relative to representative D/Yama2019 strains, 35 nucleotide substitutions were identified across the *P3* coding region, of which most were synonymous. Only eight amino acid substitutions were detected (A77T, T194K, P281S, R343K, F480Y, R516K, R569K, and G649R), indicating limited amino acid variability and overall conservation of the polymerase protein. Several substitutions were shared with Yamagata-related viruses, whereas others appeared less common and may represent regional molecular signatures.

A direct comparison of the Kazakhstan strains revealed minimal genetic variability, with only a single conservative amino acid substitution observed. Key polymerase-associated motifs, including the catalytic KDD motif and adjacent active-site residues, were conserved in both viruses. These findings collectively suggest a high degree of functional conservation of *P3*, indicating that Kazakhstan IDV are predominantly under purifying selection and retain molecular features characteristic of contemporary D/Yama2019-like strains.

The results provide crucial molecular evidence of the ongoing circulation of Asian Influenza D virus lineages in Kazakhstan, underscoring the need for sustained genomic surveillance in regional livestock populations.

## 4 Discussion

The present study provides the first molecular evidence of IDV circulation among livestock animals in Kazakhstan, thereby extending the known geographic distribution of the virus into Central Asia. PCR-positive animals were primarily cattle, which is consistent with the established role of bovines as the principal reservoir host of IDV.

The detected viral RNA prevalence (1.27%) is comparable to recent reports from other Asian countries including Pakistan (2.4%) (6), and northeastern China (1.2%) but substantially lower than the estimates from other regions of China (11.1%) (9), and the United States (up to 18%) (17). However, molecular prevalence alone is unlikely to reflect the true extent of virus circulation. Serological investigations conducted in Italy and China have demonstrated IDV antibody prevalence of up to 80–91% in cattle despite relatively low rates of viral RNA detection, indicating widespread previous exposure even when active infection is infrequently detected (27, 28).

Complete *HEF* sequences were obtained from two cattle samples collected in the Zhetysu Region. Both viruses clustered within the D/Yama2019 lineage and shared more than 98.83% nucleotide identity with contemporary members of this lineage. The Kazakhstan sequences were most closely related to the Australian NSW/L13 sequence and were positioned within a broader clade containing viruses reported from Japan, Türkiye, China and the South Korea (3, 16, 29). Recent studies have demonstrated the emergence and predominance of the D/Yama2019 lineage in East Asia, suggesting ongoing geographic expansion of this genetic lineage (3, 30). The detection of D/Yama2019-lineage viruses in Kazakhstan may reflect the broader westward distribution of this lineage across Eurasia. Expanded surveillance is particularly warranted in regions bordering Russia and China and at major livestock markets, quarantine facilities and animal-transport corridors. The identification of D/Yama2019-lineage viruses in Kazakhstan has broader implications for regional disease surveillance and biosecurity (5).

IDV evolution is characterized by relatively rapid genetic diversification and a higher nucleotide substitution rate compared with Influenza C virus. Mutational variation within the *HEF* glycoprotein plays a central role in determining antigenic characteristics and lineage differentiation (31). In particular, amino acid substitutions located within the apex region of the *HEF* protein (positions 210–214) have been recognized as important lineage-associated markers (15).

The molecular characteristics of the Kazakhstan IDV strains support their phylogenetic affiliation with the emerging D/Yama2019-like lineage currently circulating in East Asia (3). Conservation of the apex motif together with overall *HEF* sequence similarity indicates that these viruses are evolutionarily distinct from the historically predominant North American D/OK and D/660 lineages (16). Similar lineage replacement patterns have recently been reported in Japan, China, and Australia, suggesting ongoing geographic expansion of Yamagata-related IDV across Eurasia (32).

The minimal amino acid variability observed among the Kazakhstan strains suggests a relatively recent introduction followed by local transmission with limited genetic diversity.

The negative virus isolation trials in MDCK and MDBK cell lines are consistent with previous reports describing IDV’s preferential tropism for the upper respiratory tract and the need for specific host-cell adaptations to achieve efficient *in vitro* replication (4, 33).

Particular attention should be paid to detecting IDV RNA in farmed Altai Maral Deer. Although cattle are widely recognized as the principal reservoir host of IDV (34), the identification of viral RNA in clinically healthy Maral Deer suggests this species may also be exposed to the virus under natural field conditions. Shared farming environments and overlapping grazing areas with cattle probably create clear opportunities for interspecies contact and pathogen transmission. Because the viral loads in the positive deer samples were low (Ct values >34), sequencing was unsuccessful, and the genetic relationship of these viruses to the cattle strains could not be determined. Consequently, the source of infection and transmission pathways remain unclear. Nevertheless, these findings warrant further investigation of the potential role of Cervids in the ecology and maintenance of IDV in Central Asia. Previous studies have documented infection or exposure in swine, horses, camels, and wild boars (4, 35–37). The detection of IDV RNA in farmed Maral Deer warrants further investigation into possible interspecies exposure. The detection of the virus in Maral Deer also supports the hypothesis that IDV can infect novel hosts under conditions of close contact with domestic livestock and exhibits considerable ecological plasticity (38).

Given Kazakhstan’s extensive livestock sector and its strategic position connecting East Asia, Russia, and Europe, understanding these dynamics is vital within a broader One Health framework. Despite the absence of confirmed human clinical cases, IDV is increasingly regarded as a potential zoonotic pathogen (39). High seroprevalence among individuals occupationally exposed to livestock, including farmers and veterinarians, has reached up to 91% in some studies (40). Moreover, the ability of IDV to bind human respiratory receptors (9-*O*-acetylated sialic acid) and to be transmitted efficiently via aerosol routes in ferret and guinea pig models highlights its potential public health relevance (41). These findings emphasize the importance of incorporating IDV surveillance into Kazakhstan’s veterinary and public health monitoring systems within the broader One Health framework.

## 5 Conclusion

This study provides the first molecular confirmation of Influenza D virus circulation in Kazakhstan, filling an important gap in global epidemiological knowledge and confirming that Central Asia forms part of the worldwide circulation network of this emerging pathogen.

A relatively low RNA positivity rate (1.27%; 11/867 samples) was identified, consistent with reports from other Asian regions, including northeastern China (1.2%) and Pakistan (2.4%).

Notably, IDV was detected not only in cattle but also in Maral Deer, supporting the broad ecological adaptability of the virus and its capacity to involve wild ruminants in transmission cycles, as previously reported for wild boars and other wildlife species.

Phylogenetic and molecular analyses demonstrated circulation of the D/Yama2019 lineage in Kazakhstan. The Kazakhstan strains exhibited high nucleotide identity (>98%) with Asian isolates collected during 2021–2022, indicating close evolutionary relationships with currently circulating East Asian viruses.

The detection of the D/Yama2019 lineage in Kazakhstan, paralleling its emergence and expansion in China after 2021, suggests the possibility of shared epidemiological links across Eurasia. In this context, the primer panel developed in this study is a valuable molecular tool that will facilitate genomic sequencing, full-length gene recovery and continuous surveillance of IDV strains in the region.

Although IDV was not detected in pigs or camels within the present study, international evidence demonstrating high seroprevalence among livestock-exposed humans highlights the potential zoonotic significance of this virus (42). To mitigate the risk of introducing IDV, preventive strategies should prioritize supply chain traceability and targeted, risk-based molecular screening of imported livestock, paying particular attention to animals that are subclinically infected. Following the detection of IDV, integrated genomic and epidemiological investigations should be promptly initiated to characterize transmission pathways and guide measures to prevent further dissemination.

Our findings emphasize the potential benefits of providing veterinary services responsible for animal import control with molecular diagnostic kits for IDV screening and detection (43). Detection of IDV in Kazakhstan therefore underscores the need to integrate this pathogen into national surveillance programs to improve risk assessment and strengthen preparedness within a One Health context.

### 5.1 Limitations

Several limitations of the present study should be considered when interpreting the findings. First, sampling intensity differed substantially across host species (*n* = 611 cattle, *n* = 175 camels, *n* = 51 red deer and *n* = 30 pigs), which restricts direct statistical comparisons of prevalence rates between animal populations. Second, low viral loads in several positive specimens prevented complete genome amplification. Consequently, complete coding sequences were successfully recovered for the HEF and P3 gene segments from only two bovine samples, while the length of the partial sequences of PB1 and PB2 was insufficient for robust phylogenetic analysis. The incomplete recovery of all viral segments prevented a comprehensive whole-genome comparison and assessment of potential reassortment events.

Thirdly, attempts to isolate the virus in cell culture were unsuccessful, limiting further phenotypic and biological characterization of the Kazakhstan strains.

Finally, serological testing was not performed, limiting our ability to evaluate previous exposure or estimate the true population-level circulation of IDV in Kazakhstan.

Future studies integrating molecular, serological and genomic approaches across a broader and more balanced range of host species are necessary to fully elucidate the epidemiology and transmission dynamics of IDV in Central Asia.

## Data Availability

The sequence data generated in this study have been deposited in the GenBank database under the following accession numbers: HEF gene segment: PZ440420-PZ440421, and P3 gene segment: PZ712063-PZ712064.
